# Quarantine Conundrums

**Published:** 1881-07

**Authors:** 


Quarantine Conundrums.—Smallpox prevails to some extent, and has so prevailed for some time past, in Chicago, New York and Philadelphia. Every day brings us a lot of passengers by rail from those cities. Can any one tell us why not a word is said about quarantining those passengers, whilst others coming by sea from an opposite direction are scarcely permitted to land even after a long quarantine? Is the habitual cackling of some people on this subject honest or affected? If they were honest, would not alarmists show more concern than they do in the application of the only sure protective, vaccination?

There are, in all probability, 5,000 persons in San Francisco, a large proportion of whom are children, who have never been vaccinated—two or three times that number who have never been properly vaccinated—and still a much larger number in whom the protective influence of proper vaccination is more or less impaired by lapse of years. All these are food for smallpox and a source of much more danger than arrivals from infected places.—*Pacific Medical and Surgical Journal.*

